# Self-expanding or balloon-expandable TAVR in patients with a small aortic annulus: A review

**DOI:** 10.34172/jcvtr.025.33360

**Published:** 2025-12-17

**Authors:** Abeel Naseer, Muhammad Imtiaz, Muhammad Adnan Zaman, Rabia Zulfiqar

**Affiliations:** ^1^Royal Devon and Exeter Hospital, Exeter, UK; ^2^Imperial College Healthcare NHS Trust, Charing Cross Hospital, London, UK; ^3^Conemaugh Memorial Medical Center, Johnstown, PA, USA; ^4^Benazir Bhutto Hospital, Rawalpindi, PK

**Keywords:** Transcatheter aortic valve replacement, Balloon-expandable valve, Self-expanding valve, Small aortic annulus, Aortic stenosis, SMART trial

## Abstract

Transcatheter Aortic Valve Replacement (TAVR) has revolutionized the treatment of severe aortic stenosis, particularly in patients at intermediate or high surgical risk. However, valve selection in individuals with a small aortic annulus remains a clinical challenge. Comparative data on self-expanding (SE) and balloon-expandable (BE) valves are limited, and recent evidence has focused on identifying the optimal prosthesis for this subgroup. This review critically evaluates the comparative outcomes of SE and BE valves in patients with small aortic annuli, emphasizing findings from the *Small Annuli Randomized to Evolut or SAPIEN Trial (SMART)* and contextualizing them with evidence from major trials including CHOICE, SOLVE-TAVI, SCOPE I, and FRANCE-TAVI. The SMART trial demonstrated that SE valves were non-inferior to BE valves for the composite endpoint of death, disabling stroke, or heart failure rehospitalization at 12 months, while achieving superior valve hemodynamics. SE valves were associated with lower mean gradients, larger effective orifice areas, and reduced rates of prosthesis–patient mismatch and bioprosthetic valve dysfunction. Other clinical studies have shown that BE valves may offer greater procedural precision, better positioning, and lower rates of paravalvular regurgitation. Both SE and BE valves represent effective options for TAVR in patients with small aortic annuli. SE valves provide improved hemodynamic performance, whereas BE valves may offer procedural advantages. Valve selection should be individualized based on anatomical characteristics and operator experience. Long-term studies are required to assess valve durability and late clinical outcomes.

## Introduction

 Valvular heart diseases (VHD) represent an increasingly significant global health burden, particularly affecting the aging population who are afflicted with calcific aortic valve disease (CAVD). In the year 2017, it was estimated that approximately one hundred thousand fatalities were attributed to CAVD, with a global prevalence of 12.6 million cases reported during the same time period.^1^ It is noteworthy that nonrheumatic valvular diseases account for approximately 2.5 million disability-adjusted life years (DALYs), constituting 0.10% of the overall health loss attributed to all diseases. The American College of Cardiology and the American Heart Association underscore the importance of early intervention for severe VHD to enhance patient outcomes.^2^ Surgical aortic valve replacement has traditionally been considered the gold standard; however, since the pioneering transvenous valve replacement/implantation (TAVR or TAVI) performed by Dr. Cribier in 2002, TAVR has gained considerable acceptance and popularity. Presently, TAVR is indicated for select populations aged sixty-five years or older, characterized by severe symptomatic aortic stenosis (aortic valve area < 1.0 cm^2^, mean gradient ≥ 40 mm Hg, or peak aortic jet velocity ≥ 4.0 m/s), those at intermediate or high surgical risk, as well as patients identified as having prohibitive surgical risks due to comorbid conditions.^2^

 The Transcatheter Aortic Valve Replacement (TAVR) procedure has witnessed substantial advancements over the past two decades. Nonetheless, there exists a paucity of clinical data comparing the performance outcomes of self-expandable supra-annular valves (SEVs) and balloon-expandable valves (BEVs).^3-5^ The Small Annuli Randomized to Evolut or SAPIEN Trial (SMART) represents a pioneering study aimed at assessing the clinical outcomes and valve performance of SEVs and BEVs in patients undergoing TAVR for severe symptomatic aortic stenosis with a small aortic annulus measuring 430 mm^2^ or less^6^. This article will review the findings and clinical implications of the SMART trial.

## Smart Trial

###  Eligibility Criteria

 Patients presenting with severe aortic stenosis, characterized by a small annular diameter and a valve area of ≤ 430 mm^2^, who exhibited symptoms attributable to severe aortic stenosis and possessed anatomies suitable for the Medtronic Evolut PRO/PRO + and Edwards SAPIEN 3/3 Ultra devices, were eligible for enrollment. Conversely, patients with anatomies deemed unsuitable for either valve device, or those with other significant comorbidities that might preclude the safe execution of Transcatheter Aortic Valve Replacement (TAVR), were excluded from the trial. The baseline characteristics of the two groups were comparable, exhibiting no significant differences in randomization.

###  Endpoints

 The primary clinical outcome composite endpoints encompassed mortality, disabling stroke, and rehospitalization due to heart failure over a duration of twelve months. The co-primary valve function composite endpoints included bioprosthetic valve dysfunction occurring within the same twelve-month period. The parameters assessing bioprosthetic valve dysfunction comprised hemodynamic structural valve dysfunction, which is defined as a mean gradient of ≥ 20 mmHg; non-structural valve dysfunction, characterized by severe prosthesis mismatch or moderate to severe aortic regurgitation; thrombosis; endocarditis; and aortic valve re-intervention.

 The secondary endpoints encompassed hemodynamic performance as measured by mean aortic valve gradient and effective orifice area at twelve months, the incidence of prosthesis-patient mismatch (specifically, moderate or severe mismatch at 30 days), and safety endpoints characterized by the rates of major complications, which include paravalvular regurgitation and pacemaker insertion.

## Results

 The primary clinical outcome composite endpoint at 12 months for SEV was 9.4%, whereas for the BEV group, it was 10.6%. This finding demonstrated the noninferiority of SEV compared to BEV, with a difference of -1.2 percentage points (90% confidence interval, -4.9 to 2.5; *P* < 0.001 for noninferiority). Furthermore, the analysis of the co-primary valve function composite endpoint at 12 months indicated a significantly lower incidence of bioprosthetic valve dysfunction in the SEV group, with dysfunction rates of 9.4% for SEV versus 41.6% for BEV, resulting in a difference of -32.2 percentage points (95% confidence interval, -38.7 to -25.6; *P* < 0.001 for superiority).

 Regarding secondary outcomes at 12 months, the SEV group demonstrated a lower mean aortic valve gradient, registering 7.7 mmHg compared to 15.7 mmHg for the BEV group. Furthermore, the mean effective aortic orifice area was notably larger in the SEV group, with measurements of 1.99 cm^2^ as opposed to 1.50 cm^2^ in the BEV group. Additionally, the SEV group exhibited superior results in terms of prosthesis-patient mismatch at 30 days, with only 11.2% of patients in the SEV group experiencing this complication, in contrast to 35.3% in the BEV group (*P* < 0.001)

###  Safety of both valves

 The rates of major safety endpoints were similar in both groups.

 In summary, the SMART trial demonstrated that SEV is non-inferior to BEV regarding clinical outcomes and exhibits superior hemodynamic performance and reduced bioprosthetic-valve dysfunction in patients undergoing TAVR for severe aortic stenosis and small aortic annuli.

## Discussion

 The treatment of severe aortic stenosis has undergone a significant transformation due to the advent of Transcatheter Aortic Valve Replacement (TAVR). This innovative approach has proven to be particularly advantageous for high-risk patients presenting with complex anatomical challenges, such as a small aortic annulus. In patients with such intricate anatomy, the reduced valve area imposes distinct difficulties regarding accurate valve deployment, valve functionality, and the mitigation of complications, including paravalvular regurgitation. The choice between balloon-expandable (BE) and self-expanding (SE) valves is instrumental in influencing patient outcomes. Numerous clinical trials are currently investigating which type of valve demonstrates superior performance within this specific subgroup, with each study contributing valuable insights into the comparative efficacy of BE and SE valves. Through the critical analysis of these trial results, alongside the conclusions drawn from the “Self-Expanding or Balloon-Expandable TAVR in Patients with a Small Aortic Annulus (SMART)” study, a more comprehensive understanding of the impact of these valves on clinical outcomes emerges, particularly for cases characterized by challenging anatomical configurations

 The advantages of balloon-expandable (BE) valves compared to self-expanding (SE) valves have been clearly articulated in the SMART trial; these findings were consistently corroborated in numerous other clinical trials, particularly among patients presenting with complex anatomical challenges such as small aortic annuli. A statistically significant higher device success rate for the BE valve was demonstrated in the CHOICE Trial, highlighting their superior precision in valve deployment, which is of paramount importance for patients with small aortic annuli.^7^ Similarly, the FRANCE-TAVI Registry reported lower rates of paravalvular regurgitation and in-hospital mortality in instances where BE valves were employed.^8^ These outcomes are particularly significant in the context of patients with small aortic annuli, where accurate valve positioning and effective sealing are essential to mitigate complications. This trend was further substantiated by the SCOPE I trial, which indicated improved outcomes concerning mortality, stroke, and valve dysfunction.^9^

 The SOLVE-TAVI trial revealed no significant differences in composite outcomes between balloon-expandable (BE) and self-expanding (SE) valves, which suggests a degree of equivalence among intermediate- to high-risk patients. However, this conclusion may be less relevant for individuals with small annuli, as the anatomical considerations typically necessitate the more controlled expansion and tighter sealing afforded by BE valves. Table 1 illustrates the salient findings of different studies conducted to evaluate different valves in transcatheter aortic valve replacement.

**Table 1:** Multiple prior trials evaluating different valves in patients undergoing transcatheter aortic valve replacement.

**Trial**	**Patient’s Population**	**Sample Size (n)**	**Device**	**Primary Endpoint**	**Results**	**Follow-Up duration**
CHOICE^7^ 2020	High-risk	241	BE: S-XT SE: CV	Device success	BE: 95.9% vs. SE: 77.5% (P < 0.001)	5 yrs
SOLVE-TAVI^5^ 2020	Intermediate- to high-risk	447	BE: S-3 SE: Evo-R	Composite of death, CVA, AR, PCM at 30 days	BE: 26.1% vs. SE: 28.4% (P = 0.04)	1 yrs
SMART^6^ 2024	Severe aortic stenosis, small annulus	716	BE: Evo PRO + SE: S-3 ultra	Composite of death, CVA, HF, valve dysfunction, rehospitalization	SE: 9.4% vs. BE: 41.6% (P < 0.001)	12 mo
FRANCE-TAVI registry^8^ 2022	High-risk	7820	BE: S-XT / S-3 SE: CV / Evo series	mod - sev paravalvular regurgitation or in-hospital mortality	Higher regurgitation and mortality with SE (P < 0.001)	2 yrs
Compare-TAVI Trial^10^ 2024	Broad patient population	739	BE: S-3 SE: Myval	Non-inferiority trial	Non-inferiority: P = 0.85	1 yr
SCOPE I Trial^9^ 2023	Increased surgical risk	739	BE: S-3 SE: ACURATE neo	Composite of death, CVA, bleeding complications, valve dysfunction	BE: 16% vs. SE: 24% (P = 0.015)	1 mo

BE: Balloon-expandable valve, CV: CoreValve, Evo PRO + : Evolut PRO/PRO + /FX, Evo-R: Evolut R, MACCE: Major adverse cardiac and cerebrovascular events, SE: Self-expanding valve, TAVI: Transcatheter aortic valve implantation S XT: Sapien XT, S-3: Sapien 3, S-3 ultra: SAPIEN 3/3 Ultra

 In conclusion, although SE valves may demonstrate satisfactory performance in specific populations, the aggregate evidence derived from these trials indicates that BE valves typically provide enhanced performance in patients with small aortic annuli. These valves ensure more dependable deployment, superior long-term durability, and reduced incidences of adverse outcomes such as paravalvular regurgitation and mortality. Such factors are crucial in the management of the intricate anatomy associated with these patients.

 The comparatively limited sample size may constrain the generalizability of the results and diminish the statistical power necessary to identify clinical differences. Additionally, another factor affecting the generalizability of the trial is the inclusion of patients with small aortic annuli, which implies that the findings may not be applicable to patients with larger valve areas or varying anatomies. Furthermore, the trial only evaluated two types of Transcatheter Aortic Valve Replacement (TAVR) devices, namely, the Medtronic Evolut PRO/PRO + and the Edwards SAPIEN 3/3 Ultra, which may influence the relevance of the findings in light of the swift advancements in TAVR technology. Moreover, the differences in valve design may also introduce inherent biases in the outcomes, such as hemodynamic performance and prosthesis-patient mismatch, thus necessitating cautious interpretation.

 Furthermore, the twelve-month follow-up interval may prove insufficient for the evaluation of long-term outcomes, including valve durability and the emergence of late complications. The primary endpoint, which is a composite of multiple outcomes, may obscure the importance of the individual components. In addition, the lack of detailed criteria for defining a small annulus could result in variability in patient selection. The exclusion of patients with significant comorbid conditions restricts the applicability of the trial to a broader population with more complex comorbidities. Finally, while improved hemodynamic outcomes have been observed with SEV, the long-term clinical significance of these findings remains uncertain.

## Conclusion

 Transcatheter Aortic Valve Replacement (TAVR) has become an established therapy for severe aortic stenosis, particularly in patients at increased surgical risk. However, managing patients with a small aortic annulus remains complex due to anatomical constraints that affect valve performance. Evidence from the SMART trial and other major studies indicates that both self-expanding (SE) and balloon-expandable (BE) valves are safe and effective in this population. SE valves provide superior hemodynamic outcomes, including lower transvalvular gradients and reduced prosthesis–patient mismatch, while BE valves offer advantages in deployment accuracy and paravalvular sealing. Valve selection should therefore be individualized according to patient anatomy, procedural feasibility, and operator experience. Continued research with longer follow-up and evolving device technologies is essential to better define durability, optimize valve design, and improve long-term outcomes for patients with small aortic annuli undergoing TAVR.

## Competing Interests

 The authors declare that they have no conflicts of interest related to the content of this article.

## Ethical Approval

 This article is a narrative review and does not involve any studies with human participants or animals performed by the authors. Therefore, ethical approval was not required.
